# The Role of Extracellular Vesicles in the Control of Vascular Checkpoints for Cancer Metastasis

**DOI:** 10.3390/cancers17121966

**Published:** 2025-06-12

**Authors:** Fang Cheng Wong, Janusz Rak

**Affiliations:** The Research Institute of the McGill University Health Centre, Montreal, QC H3A 0G4, Canada; wong.wongfangcheng@mail.mcgill.ca

**Keywords:** extracellular vesicles, exosomes, particles, angiogenesis, metastasis, vasectasia, vascular cooption, coagulation system

## Abstract

Systemic cancer progression culminating in metastatic disease is implicitly dependent on tumour cell interactions with the vascular system. Indeed, different facets of the micro- and macro-vasculature can be regarded as rate-limiting ‘vascular checkpoints’ in the process of cancer dissemination. The underlying complex communication networks drive tumour neovascularization, angiogenesis, immunoregulation, activation of the coagulation system, angiocrine interactions, and non-angiogenic vascular responses across multiple cancer types. Yet, each cancer may represent a unique vascular interaction scenario raising a prospect of targeted modulation of blood and lymphatic vessels for therapeutic purposes, beyond the traditional notion of tumour anti-angiogenesis. While the emphasis of studies aiming to understand this circuitry has traditionally been on soluble, or ‘mono-molecular’ mediators, the rise of the particulate secretome encompassing heterogeneous subpopulations of extracellular vesicles (EVs; including exosomes) and particles (EPs) brings another dimension into the tumour–vascular communication web during the process of metastasis. EVs and EPs are nanosized cellular fragments, the unique nature of which lies in their ability to encapsulate, protect and deliver to target cells a range of bioactive molecular entities (proteins, RNA, DNA) assembled in ways that enable them to exert a wide spectrum of biological activities. EVs and EPs penetrate through biological barriers and are capable of intracellular uptake. Their emerging vascular functions in metastatic or infiltrative cancers are exemplified by their roles in pre-metastatic niche formation, thrombosis, vasectasia or angiocrine regulation of cancer stem cells. Here, we survey some of the related evidence supporting the biological, diagnostic and interventional significance of EVs/EPs (EVPs) in disseminated neoplastic disease.

## Simple Summary

Cancer metastasis involves multiple contacts between cancer cells and the vascular system. The web of underlying, rate-limiting and reciprocal tumour–vascular interactions acts as a series of metastatic checkpoints regulating cancer cell entry into the blood stream, dispersion into remote organ sites, conditioning of distant tissues for metastasis, triggering of structural and functional changes in blood and lymphatic vessels, immunoregulation, formation of vascular niches for clonogenic cancer cells and many other aspects of the metastatic process. Among the effector mechanisms of tumour–vascular crosstalk, the release and intercellular trafficking of cellular fragments known as extracellular vesicles and particles (EVPs) emerged as a unique form of cell–cell communication. EVPs serve as hubs for regulated assembly of molecular packets, enabling their unconventional secretion and transmission between cells. Their cargo consists of proteins, lipids, RNA and DNA including oncogenic, regulatory and biologically active mediators. In this manner, EVPs impact multiple vascular facets of metastasis, ranging from angiogenesis, blood coagulation and formation of pre-metastatic niches (PMNs) to disruption in endothelial cell lining and changes in vascular permeability. Understanding how different EVP subsets contribute to vascular checkpoints in specific cancers may enable developing tailored approaches to predict, prevent or oppose their pro-metastatic activities.

## 1. Introduction—The Pivotal Role of the Vasculature in Systemic Manifestations of Cancer

Although cancer may initially emerge as an apparently localized lesion marked by circumscribed alterations in cellular architecture, phenotype and genetic make-up, it is the subsequent multifocality and dissemination that renders the disease intractable [1]. This is not necessarily an inevitable scenario, but if untreated, many cancers evolve, progress and metastasize regionally and systemically, with a dramatic increase in mortality and precipitous diminution of curative opportunities [2].

The fulcrum in the transition from the local to systemic, life-threatening malignancy lies with the involvement of the vascular system. The vasculature interconnects organs, tissues, cellular neighborhoods and biological compartments, and thereby it exerts potent integrative and regulatory effects across the body’s cellular ecosystems [3]. This fundamental physiological role is dramatically subverted in cancer. While overt metastatic dissemination is the most obvious manifestation of the vascular dependency in the course of cancer progression, there are multiple lines of evidence to suggest that cancer-related, long-range, systemic perturbations may also occur irrespectively of physical cancer cell spreading [4], and sometimes emerge prior to, or in parallel with, overt dissemination [5].

The roles of blood vessels and lymphatics are central to systemic effects of cancer, beyond their function as metastatic conduits [4,6]. Tumour–vascular interactions include systemic immunomodulation and immunosuppression, perturbations within the hemostatic system (cancer coagulopathy), remote inflammatory responses [6] and metabolic alterations, such as cachexia [7], glucose intolerance [8], or hepatic steatosis [9], along with a wide spectrum of more specific paraneoplastic syndromes with endocrine, neurological, cutaneous and other manifestations [10,11]. These pathologies contribute to the overall morbidity in cancer patients, while in some cases may be directly or indirectly involved in conditioning of tissues for (or against [6,12]) metastatic colonization and therapeutic failure [4]. The totality of regulatory influences that the vasculature may exert upon the local and systemic hallmarks of metastatic cancer could be regarded as a network of ‘vascular checkpoints’ (beyond the use of this phrase in immunotherapy [13]), signifying the notion that these blood vessel-dependent processes could be therapeutically modulated beyond the paradigm of anti-angiogenesis.

## 2. Multifaceted Involvement of the Vasculature in Systemic Cancer Progression

Both macro- and microcirculation contribute to systemic hallmarks of cancer, albeit in markedly different ways. Tumour microvasculature acts as a multifunctional ‘organizer’ of the tumour microenvironment (TME), a blood supply system reaching individual cells, a barrier regulating the exchange between circulating blood and the TME, and a source of paracrine (angiocrine) regulation of cells in cancer parenchyma and stroma [14,15]. General circulation extends these localized effects across multiple body systems, mediates long range effects and is a site of certain systemic morbidities (e.g., thrombosis) leading to formation of cancer-associated, pathological macro-environments [16].

These various processes result in a multi-layered vascular dependence of metastatic progression. For example, microscale cancer cell survival, regardless of site, is critically dependent on the proximity to the nearest patent capillary, usually within less than 200 μm due to the limits of oxygen and nutrient diffusion gradients [17]. Blood vessels regulate the levels of tumour hypoxia and access to blood-borne sources of energy, hormones, drugs and immune cells [3]. The endothelial cell secretome acts as a central element in the angiocrine regulation of cancer cell behaviour [14,15,18,19], including formation of perivascular cancer stem cell niches that mold the properties and activities of tumour-initiating cells [20,21]. It could be speculated that vascular contact may often represent a part of cancer cell stemness [22].

Blood vessel walls form a selective barrier that controls the emission of cancer-related signals into the blood stream (cytokines, factors, cell free DNA, and extracellular vesicles) and regulates the pool of circulating tumour cells (CTCs) and their pro-metastatic clusters entering the systemic circulation [22,23,24], features that may be altered by various degrees of vascular permeability [25]. The latter property is not only responsible for the leakage of plasma into the TME, but may also control the trafficking of immune cells [26], self-seeding of cancer cells, and the levels of tumour exposure to systemic anticancer therapies [27]. Recycling of interstitial fluid through the lymphatic vasculature enables trafficking of cancer cells and their products into regional lymph nodes [28], while the formation of tertiary lymphoid structures (TLSs) around high endothelial venules within and around the tumour mass further regulates the traffic and accumulation of immune cells within the TME [29]. Naturally, these crucial processes are not executed by default, but instead, they are a function of regulatory milieu unique to specific disease contexts.

Events occurring within the systemic macrocirculation also markedly affect cancer progression. In addition to physically connecting sites of primary and metastatic cancer growth, macro-circulation is a gateway to all systemic events associated with cancer progression. For example, cancer-associated thrombosis (CAT) may have its biological triggers within the tumour mass [30], but the main sites of the related thrombotic morbidity are peripheral large vessels at a distance from the tumour location, either in extremities (deep vein thrombosis), or in the lung macrocirculation (as pulmonary embolism) [31]. In certain settings, thrombi may also occur across peripheral microcirculation (as disseminated intravascular coagulation) [32]. CAT is not only morbid in its own right, but also correlates with poor prognosis and therapy resistance, and it often intensifies in metastatic cancers [33].

Many cancer-associated vascular events have multifactorial consequences. For example, the expansion of the tumour microvasculature visibly affects formation of upstream larger tumour ‘feeding vessels’ [34]. Cancer-related perturbations in endothelial barrier function, including induction of vascular permeability [25], or endothelial damage [35] influence the passage of cells and molecules between systemic circulation and the TME. The release of cytokines provokes alterations in the repertoire of endothelial adhesion molecules, a process that may lead to immune ‘anergy’ associated with some cancers [26]. These processes may also be exacerbated by systemic deregulation of immune cell mobilization and trafficking and many are instigated by blood-borne cancer cell products [36]. In brain tumours, for example, the compounded effects of residual elements of the blood–brain barrier (BBB) and the emerging brain–tumour barrier (BTB) create unique conditions for immune cell exclusion, a process that impedes the effects of systemic immune surveillance and immunotherapy, along with its negative impact on chemotherapy and other treatments in this class of malignancies [25]. In the circulating blood, the activated coagulation system and platelets may envelop CTCs, thereby preventing their immune destruction while facilitating their role in metastasis [37]. These and other examples highlight the link between systemic tumour progression and the evolving roles of the vascular system components, as reviewed extensively in the recent literature [4,15,25].

## 3. Cancer-Associated Vascular Pathologies—Angiogenesis and Beyond

Neoplastic processes not only exploit vascular networks, but also profoundly modify them. This influence is often equated with the ability of cancer cells, or their surrounding TME to trigger angiogenesis [38]. It is, however, increasingly clear that vascular responses to cancer progression are as diverse as cancers themselves, and they may extend far beyond the conceptual framework of the canonical angiogenic vascular growth program [38,39,40,41]. Mapping this program since the early 1990s has exerted enormous stimulating influence on biological paradigms developed around the tumour–vascular interface. They have also been very influential in terms of designing blood vessel-modulating therapies in cancer, especially the concept of anti-angiogenesis [3,42,43].

The lens with which tumour neovascularization has frequently been perceived was shaped by insights from developmental blood vessel-forming processes [44]. In this context, the expansion of new capillaries from pre-existing vascular structures leads to formation of new blood vessel ‘units’, through a process often referred to as angiogenic sprouting [38]. The sprouting mechanisms can be reactivated postnatally during reparative and wound healing processes [38], and play an important (albeit not exclusive) role in pathological vascular growth, including in cancer [41]. Under these conditions, sprouting angiogenesis is often triggered by a localized, hypoxia-driven upregulation of vascular endothelial growth factor (VEGF), a multifunctional dimeric polypeptide, which forms a diffusion gradient able to attract the coordinated vascular responses in a form of directional outgrowth of finger-like endothelial cell cohorts (sprouts) [38,45]. Sprouts are composed of specialized VEGF gradient-seeking endothelial tip cells, followed by columns of dividing stalk endothelial cells, structures extending from the pre-existing vessels toward the stimulus [39]. Normally, the subsequent lumen formation, anastomosis with nearby vessels, resumption of blood flow and recruitment of pericytes complete the formation of a new capillary loop, and lead to tissue reoxygenation, thus terminating the hypoxic trigger of sprouting [38]. These complex responses require the orchestrated contribution of several mediators and their receptors (VEGFR2, NOTCH, TIE2, PDGFRb), a molecular network that governs interactions between endothelial cells, pericytes and myeloid cells participating in the angiogenic growth processes, as extensively reviewed elsewhere [38,40,41,46].

In cancer, elements of the angiogenic program may be highjacked and dysregulated, leading to the formation of aberrant, dysmorphic, leaky and dysfunctional microvascular structures [3,38,43,47]. These altered responses may result in regional hypoperfusion, hypoxia, necrosis, and tumour micro-thrombosis [25,48,49], along with corresponding responses of extra-tumoural supply/feeding vessels [34,50]. The imbalances of angiogenic stimulators and inhibitors, chronic activation of their upstream regulating pathways, or protracted exposure to proangiogenic TME are among the triggers implicated in these vascular anomalies [25]. The important feature of these processes is also the frequent upregulation of VEGF in cancer [42], resulting from microenvironmental hypoxia [45], or chronic inflammation [51]. In addition to microenvironmental factors, tonic oncogenic signalling cues drive VEGF upregulation and the pro-angiogenic cancer cell secretome [41,52].

## 4. Alternative Neovascularization Pathways in Cancer

While the core angiogenesis program and activation of VEGF-driven pathways are frequently a part of the tumour neovascularization process, multiple alternative, often context-specific mechanisms are also at play [41]. This is important to consider as the introduction of VEGF pathway inhibitors into anticancer treatment regimens led to lasting and important, but hardly universal, improvements in outcomes [15,42,53]. This observation suggests that, in addition to (or instead of) VEGF, multiple other mediators may contribute to vascular growth and remodelling within the vasculature supplying the tumour mass [25] and at metastatic distant organ sites [54], often acting in ways that may not involve canonical pathways of angiogenesis. Indeed, multiple non-angiogenic neovascularization mechanisms have already been identified, and extensively reviewed in the recent literature [41,53,55,56]. Among them, some of the more studied examples include processes of vascular co-option [53], postnatal vasculogenesis [38], vasculogenic mimicry of cancer cells [57], intussusceptive angiogenesis [58], tumour arterio-veno-genesis [34,50], vascular dysmorphia [59], vasectasia [60] and several others already extensively described [41]. In addition, the onset of lymphangiogenesis in and around the tumour mass and within regional lymph nodes, along with remodelling of connecting lymphatic vessels and other effects, may contribute to metastatic dissemination and play a role in vascular system conditioning for regional and systemic cancer progression [61,62,63].

The cancer-driven vascular responses are also reflected in the heterogenous landscapes of vascular cell phenotypes that emerge in the course of cancer progression. For example, recent insights from single-cell sequencing studies revealed major rearrangements within the vascular compartment [60,64,65,66,67]. These studies point to a remarkable heterogeneity amongst tumour-associated endothelial cells, including phenotypes harbouring a high degree of cancer specificity. Such idiosyncratic endothelial cell subtypes (or states) have been reported in lung [67], brain [60,65] and breast cancer settings [68], and are likely to also exist elsewhere. It would be of great interest to ask whether endothelial, mural or perivascular regulatory cells in pre-metastatic organ sites or at sites of overt metastasis also undergo similar (or different) cellular changes, and what are the related mechanisms and mediators. The totality of these responses is still poorly understood, but their analysis may open new paths to therapeutic interventions in metastatic cancer, beyond the realm of the traditional anti-angiogenic therapy [15].

## 5. Vascular Checkpoints During Metastatic Dissemination

Since various elements of the vascular system (angiogenic blood vessels, coagulation system, circulating immune cells) play rate-limiting roles in various aspects of metastatic dissemination, they can be regarded as modifiable vascular checkpoints of considerable therapeutic interest (again, beyond traditional anti-angiogenesis) [41]. Those vascular regulatory nodes could exist both amidst the global hallmarks of the metastatic process or be more nimble and context-specific.

**Figure 1:**
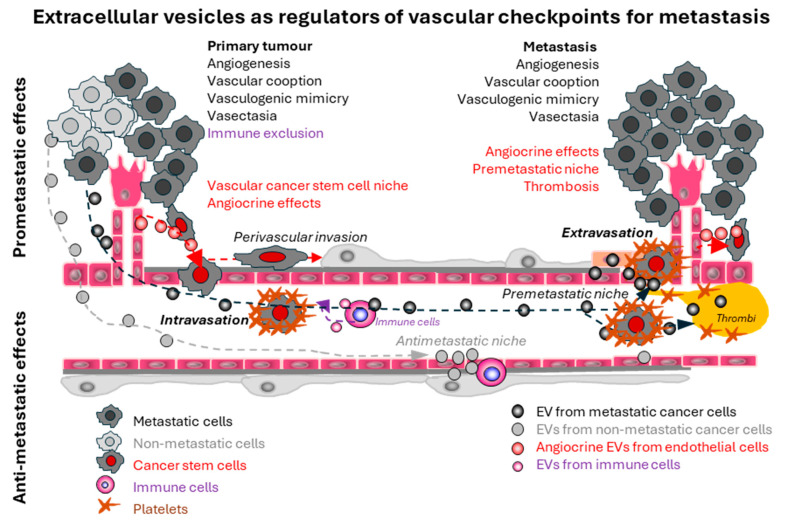
Examples of the multiple roles that extracellular vesicles play in the control of vascular checkpoints during the multistep process of cancer metastasis. Extracellular vesicles (EVs) are involved in all vascular processes (checkpoints) and steps of the metastatic cascade from angiogenesis in the primary tumour to non-angiogenic vascular responses, and from angiocrine stimulation of cancer stem cells to the formation of pre-metastatic niches and establishment of macroscopic secondary tumours. While cancer stem cells, highlighted here with red nuclei, are not the only cells capable of intra- and extravasation, their successful passage through vascular steps of the ‘metastatic cascade’ is more likely to result in formation of secondary tumours due to their tumour initiating properties (see text for details).

The common conceptual framework within which the analysis of metastatic processes is traditionally considered is often referred to as the ‘metastatic cascade’ [1] (Figure 1). This is meant to acknowledge that cancer cells undergo multiple rate-limiting, sequential, selective and inherently inefficient transitions on their path from the primary tumour to their metastatic destinations. These steps include the departure from the tumour mass into the blood stream (intravasation), survival in blood, homing to secondary sites (often with a degree of organ tropism), extravasation into the surrounding tissue and resumption of the secondary, vascular dependent growth [1] (Figure 1). Implicitly, many of these rate-limiting steps are dependent on interactions between cancer cells and the vasculature [1]. Examples of these ‘vascular checkpoints’ include the extent to which primary tumour microcirculation facilitates the access of cancer cells to blood and lymphatic channels, intravascular processes of immune surveillance and cancer cell destruction, permissive vascular niches at sites of dissemination, and many others [1].

In addition to these global mechanisms, metastatic dissemination processes associated with specific cancers exhibit multiple idiosyncratic features with correspondingly variable vascular underpinnings. In this regard, the diversity of metastatic mechanisms may comprise variation in the temporal order of dissemination relative to progression at the primary tumour site (e.g., metachronous, synchronous, or unknown primary-type metastasis) [69,70], histological types of metastatic lesions [71,72], their genetic heterogeneity [73], tropism for different organ sites (‘soils’) [70,74,75] and variable cellular progression pathways in a given cancer setting (e.g., sequential or parallel progression at primary and metastatic sites) [69,70,75,76]. These different scenarios are paralleled (and influenced) by increasingly well documented vascular characteristics. For example, the two recently described distinctive subtypes of liver metastasis, known as replacement and desmoplastic lesions, are linked to either vascular co-option or angiogenesis, respectively [71], two fundamentally different vascular processes of great therapeutic significance and linked to unique diagnostic challenges [77].

While the vasculature is essential for the metastatic processes to occur in general (exceptions include dissemination within fluid spaces in the brain [78]), its regulatory impact may be either pro- or anti-metastatic. The latter includes vascular barriers impeding intra- and extravasation of cancer cells, immune or mechanical destruction of CTCs in the blood stream, endothelial cell-dependent mechanisms that may modulate cancer cell growth or phenotype and others [18].

Indeed, endothelial cells may modulate cancer cell state [18], dormancy [79], invasiveness [80], stemness [15,20,21,79], plasticity [81], or may be involved in tumour cell regression [82]. There are several examples illustrating these powerful influences. In a recent study, glioma cells were found to use the NOTCH signalling pathway to remain within the perivascular niche in close physical contact with the endothelium. When the NOTCH pathway was inhibited, these cells switched their growth pattern to a niche mediated by the formation of microtube connections between cancer cells themselves [81]. In another study, invading glioma stem cells were found to masquerade as pericytes, whereby they used outer surfaces of vascular channels for tissue infiltration [83]. In other cancer settings, inherent invasiveness of angiogenic endothelial cells was found to be a force stimulating migration of adjacent cancer cells [80].

Thus, as new insights emerge, the traditional view of the ‘metastatic cascade’ gives way to a more complex paradigm where cancer dissemination can be seen as a culmination of effects exerted by the systemic, pliable pro-metastatic communication network formed around the vasculature [4]. This circuitry entails short- and long-range interactions between cancer cell populations and their surroundings. One example in this regard includes communications involving metastatic ‘instigator’ cells that control dissemination of other cancer cells [84]. Other studies have shown cell–cell cooperation in metastasis [85] or regulatory effects known as ‘concomitant immunity’, whereby metastatic growth may be curtailed in the presence of a remote large tumour mass [86], potentially with the involvement of circulating angiogenic inhibitors [3]. Long-range signals from the primary tumour may also pre-condition distant organ sites for incoming metastasis, a phenomenon often described as a pre-metastatic niche (PMN) effect [54,74,87,88,89]. On the other hand, systemic effects transmitted by the vasculature may also provoke distant immune responses that may curtail impending metastatic dissemination [6,9,12].

Description of these complex relationships where different facets of the vasculature (blood vessels, plasma, hemostatic system, circulating immune cells) act as checkpoints for metastasis is often placed in the context of canonical mediators including cytokines, extracellular matrix molecules, adhesion receptors and their signalling cascades, as summarized in detail elsewhere [75]. However, over the past two decades, accumulating data revealed the existence of another layer of biological regulation. In this regard, reports suggest that the exchange of information within cellular ecosystems of aggressive cancers involves a wide range of mechanisms that depend on the formation of junctional, synaptic or tubular connections (e.g., microtubes, tunnelling nanotubes) that enable a large scale exchange of molecular content between cells, including fluxes of ions, molecules, or organelles (e.g., mitochondria [90,91]), thereby achieving a hitherto unappreciated level of functional integration [92,93,94]. An important element of this emerging circuitry also involves long-range communication via complex multimolecular structures, known as extracellular vesicles and particles (EVPs) [95,96,97]. EVPs have increasingly been implicated in fundamental processes surrounding tumour metastasis and its vascular dimensions [5].

## 6. The Rise of the Particulate Secretome

The cellular secretome has long been seen through the prism of its constituent soluble mono-molecular mediators. However, it is increasingly clear that in addition to hormones, growth factors, lipids, enzymes and other bioactive molecules, all cells also release a rich repertoire of EVPs, including more complex, particulate, sedimentable assemblies of biological effectors, hitherto often regarded as cellular ‘debris’ [98]. The term EVPs collectively describes two distinct classes of particles, of which extracellular vesicles (EVs) are cellular fragments enveloped in slivers of plasma membrane encapsulating selectively packaged molecular ‘cargo’ that includes elements of the cytoplasm, and in some cases also material from the nucleus, or cellular organelles [95,97,99]. EVs are highly heterogeneous in terms of size, subcellular origin, mechanisms of biogenesis, molecular composition and biological activity [97,100]. While these properties have been extensively reviewed in the recent literature [97], it is worth mentioning that some aspects of this diversity come, essentially, from subcellular sites of EV biogenesis, such as protrusions in the outer plasma membrane that give rise to particles often described as microvesicles (MVs), or ectosomes [96,97]. On the other hand, the intracellular membranes of the late endosome serve as sites where intraluminal vesicles (ILVs) form in a regulated manner to be subsequently released from cells as distinct small EVs (50–150 nm), also known as exosomes [95,96].

**Figure 2:**
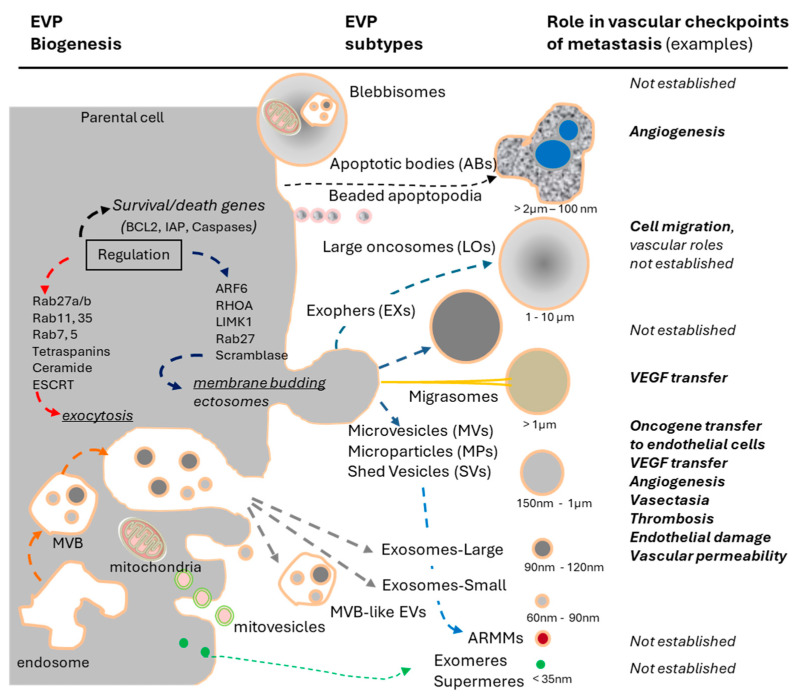
Emerging complexity of the particulate secretome in cancer and its role in tumour–vascular interactions during metastasis. A simplified diagram of subtypes, subcellular origins, properties and examples of vascular effects of EVs and EPs ([60,88,96,101,110,114,115,116,117,118,119,120]; for additional details see Table 1).

EVs exhibit diverse physical and molecular properties (Figure 2). For example, the sizes of MVs may vary dramatically from small vesicles known as arrestin domain-containing protein 1-mediated microvesicles (ARMMs; ~100 nm in diameter) to heterogenous membrane-derived EVs (>150 nm) and larger structures that may reach sizes beyond 1 μm in diameter (large oncosomes, migrasomes, exopheres, apoptotic bodies, or bebbisomes) [96,101]. Even seemingly uniform small exosome-like EVs may consist of numerous subsets of vesicles, as can be inferred from their bulk proteome, which may include upward of 1000–2000 protein signals, a number predictive of dozens of small EV subpopulations [102], given the steric constrains of individual EVs [103]. This diversity may also be detected more directly using high-resolution technologies that enable phenotyping of single EVs and their subpopulations, such as nano-flow cytometry [104,105], on-chip fluorescent imaging, super-resolution microscopy and microfluidic devices [106], or through label-free approaches including Raman spectroscopy [107], among other approaches.

In addition to proteins often indicative of the subcellular origin of different EV subsets (e.g., membrane receptors, endosomal proteins), EVs may also carry a rich cargo of nucleic acids (RNA, DNA), lipids and other bioactive material, which is either passively or actively loaded during processes of EV formation [96,108]. While in cancer, some of the EV cargo may consist of cancer-specific molecules (e.g., mutant oncogenes) [109], other molecular species may be shared with different pathological conditions. For example, microRNA-21 is packaged into EVs of several cancer cell types including glioblastoma [110], but is also a part of EV cargo in inflammation and brain injury [111].

The complexity of the particulate secretome is further compounded by recent discovery of membrane-less extracellular particles (EPs), such as exomeres [112] and supermeres [113], small structures (<40 nm) that originate from presently unclear subcellular domains and carry rich repertoires of RNA and proteins. Again, the broad repertoire of the bulk EP cargo is also suggestive of the existence of their multiple subpopulations with different properties and yet undefined functions [102].

## 7. The Emerging Biological Functions of Extracellular Vesicles and Particles

The functional roles of EVPs are only partially understood, but they collectively form a rather distinct paradigm of biological regulation [121]. Historically, the original discovery of exosomes, as carriers of transferrin receptors during erythropoiesis, implicated them as merely efficient dumping devices that cells may use for mass expulsion of their superfluous molecular content [122,123]. Formation of apoptotic vesicles/bodies by dying cells may signify another aspect of cargo disposal, such as the sequestration of cellular remnants for more efficient phagocytic removal without triggering chronic inflammation [124].

In addition to these ‘house keeping’ roles, several other basic functions of EVPs have also gradually come to light [125]. For example, EVs carrying pro-coagulant transmembrane receptors in the context of lipid membranes were suggested to act as acellular carriers of pro-thrombotic activity in various pathological states, including cancer [30,126,127]. EVs may also serve as hubs of proteases [19,128] or extracellular matrix (ECM) proteins [129], whereby they could modify extracellular microenvironment and migratory cell behaviour. EVs may also be loaded with potent soluble mediators, such as VEGF and other factors, which can be trafficked to remote locations and liberated in a bolus-like manner circumventing the limits posed by diffusion gradient formation [130].

EV formation processes also represent a unique mechanism whereby insoluble, transmembrane signalling proteins (receptors or ligands) may be released from cells and engage, on contact, with the corresponding cognate structures on surfaces of other cells, either locally or over long distances, exerting both rapid and more protracted effects [131]. Thus, EV-associated NOTCH ligand (DLL4) interacts with the NOTCH receptor on endothelial cells [132]. EV-associated integrins facilitate their homing to different organ sites enriched with corresponding ECM proteins in preparation for metastasis [74]. Similarly, cytokines associated with EV surfaces were found to activate their cognate receptors on other cells, often with a considerable increase in potency by comparison to soluble ligands [133].

EPs, such as exomeres and supermeres, are still poorly characterized in terms of their biological properties and functions. However, it is noteworthy that exomeres may possess metabolic activities [9], while supermeres are enriched for important microRNA species and carry bioactive protein cargo, such as TGFBI and other proteins, that may play a role in their biological effects [113]. EPs also have a distinct profile of biodistribution with seemingly greater ability to penetrate the brain [113]. It is very likely that multiple subpopulations of EPs may co-exist and contribute to different functions, but whether vasculature is among their main targets (or sources) is yet to be established.

**Figure 3:**
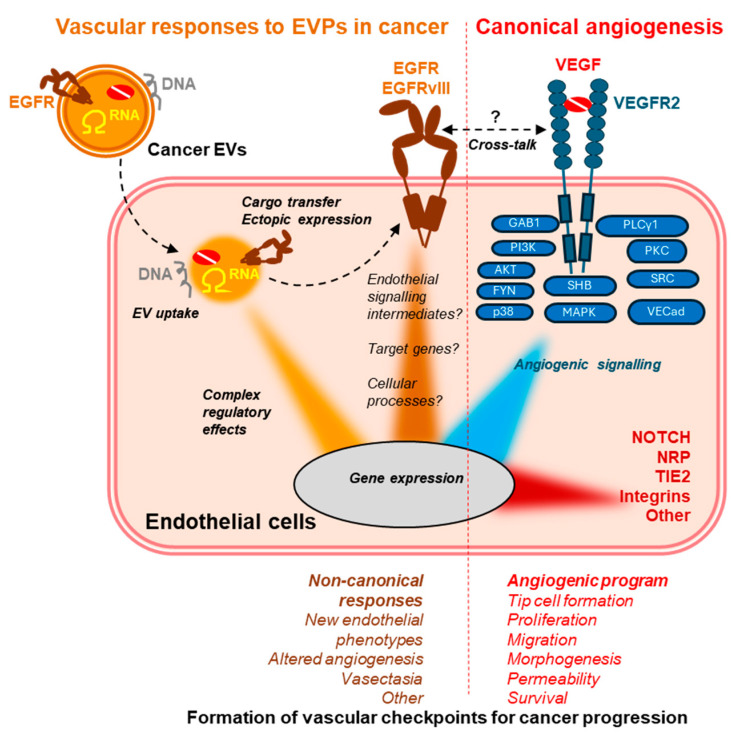
Convergence of EVP-mediated vascular regulatory pathways and the canonical angiogenesis signalling programs on tumour-associated endothelial cells. While endothelial cells are pre-programmed to respond to canonical cues triggered by VEGF, VEGF receptors (especially VEGFR2) and other physiological regulators (NRP, NOTCH, TIE2, integrins, VE-cadherin), cancer EVPs and EVPs from cancer-associated stromal cells insert additional elements into the endothelial cell signalling apparatus. For example, ectopic transfer of oncogenic EGFR may abort standard angiogenic responses and trigger alternative processes, such as vasectasia [60]. It is not clear whether such ectopic EGFR signalling involves standard molecular intermediates [144] and whether/how it intersects with the VEGF-driven angiogenesis pathway [46]. Endothelial responses are also influenced by other cargo of EVPs (RNA, DNA, proteins; see text). Collectively, these changes impact the involvement of the vasculature at different steps of cancer progression and metastasis.

Among unique functionalities associated with EVPs, one that is perhaps the most tantalizing is the ability of these particles to transfer molecular cargo between cellular populations [109,110,121,134,135]. This process is predicated on the ability of EVP to interact with the plasma membrane of recipient cells and undergo internalization, either through membrane fusion (for EVs) [134], or by endocytosis, macropinocytosis or phagocytosis. While internalization of EVPs may lead to their lysosomal degradation, there are also ample examples of their retention and re-utilization by recipient cells [109,121,136]. In this manner, different biotypes of RNA, as well as DNA and proteins, including transmembrane receptors [109], and mitochondria [137], may become incorporated into the molecular repertoire of recipient cells, altering some of their phenotypes, signalling properties and functions [121] (Figure 3). Such EV-mediated molecular exchanges are also readily documented using various reporter genes, fluorescent dyes and tagged proteins [138,139,140], and they have inspired the application of EVs as drug and gene therapy carriers [141]. Moreover, the various aspects of EV-mediated molecular transfer have been implicated in metastasis, as extensively covered in the literature [5,142,143]. Nonetheless, the emerging core function of EVPs and their subsets is the unique form of cell–cell communication, a role superimposed and intertwined with other pathways regulating collective cellular behaviour [102,121] including their roles in tumour–vascular interactions during metastasis (Figure 2).

## 8. Extracellular Vesicle Communication as Regulatory Target of Oncogenic Pathways in Cancer

While vesiculation is a conserved and common property of all cells [95], oncogenic driver events in cancer impose a level of cancer specificity on EV biogenesis, cargo and function [145]. For example, cancer cells expressing specific oncogenes (MYC, RAS, AURKA, EGFR) generate higher numbers of EVs than their isogenic non-transformed counterparts, and these EVs markedly differ in their repertoires of proteins, lipids, RNA and DNA [109,146,147,148,149,150]. For example, oncogenic transformation in glioma and colorectal carcinoma regulates the content of EV-associated procoagulant molecules [30,48,127], pro-invasive proteins [149] and microRNA [151].

In some cases, loss of tumour suppressor genes may also impact spontaneous, or stress-induced vesiculation [152]. For example, in prostate cancer cells, DIAPH3 gene loss triggers blebbing and release of micrometer-sized EVs known as large oncosomes [115,153]. Oncogene-directed targeted agents may also alter the repertoire of cancer cell-derived EVs [104,154], as does radiation [152] or induced differentiation of cancer stem cells [155]. DNA containing EVs from mutant RAS-driven cancer cells trigger signs of DNA damage response and genetic instability in recipient endothelial cells [156].

Cancer cells driven by mutant oncogenes not only vigorously produce EVs, but also often exhibit an elevated EV uptake capacity, and may switch to an alternative EV uptake mechanism, such as macropinocytosis instead of endocytosis [157,158,159]. These processes may directly impact metastatic potential of cancer cells [159].

One truly unique aspect of EVP biogenesis in cancer cells is the ability of these particles to serve as a source of ‘extracellular oncogenes’, which can be transferred between cells and exert quasi-transforming effects on their recipients [121]. For example, viable cancer cells driven by oncogenic HRAS or EGFRvIII have been shown to produce EVs carrying these very oncogenes [109,146], as either bioactive oncoproteins, transcripts or fragments of coding oncogenic DNA, including full-length sequences [146]. Oncogenic DNA sequences have also been detected in apoptotic EVs emanating from dying cancer cells [160]. Exposure of non-transformed cells to EV-associated oncogenic cargo was shown to trigger anchorage-independent growth responses and other signs resembling malignant transformation, albeit relatively transient in duration [158]. Thus, cancer progression and the accumulation of genetic and epigenetic hits creates a major shift in the molecular composition of the particulate secretome, which may contribute to events occurring at the tumour–vascular interface.

## 9. Impact of Oncogenic Extracellular Vesicles on the Tumour Vasculature

The ultimate triggering mechanism driving the formation and evolution of the TME, including its vascular components, is the succession of oncogenic alterations in cancer cells and their impact on cancer cell secretome [52]. Superimposed with influences of hypoxia, inflammation and other factors, oncogene-dependent changes in the repertoire of cancer cell-derived mediators reprogram the regulatory environment of the TME, along with interactions with blood vessels at primary and secondary cancer sites. These changes include both soluble mediators and EVPs [52,127,161,162].

In this regard, EVPs (especially EVs) that carry oncogenic cargo represent a unique category of vascular effectors due to their ability to transfer cancer-specific, mutant and potentially transforming macromolecules to other cells, including untransformed host endothelium, a process that might be expected to elicit correspondingly unusual biological responses. Indeed, EVs produced by epithelial cancer cells harbouring strongly transforming human mutant HRAS oncogenes, and enriched for genomic DNA, including full-length HRAS sequences, readily transferred these cargoes to primary endothelial cells, triggering transient proliferative responses [158], migration and signs of genetic instability [156], but no overt or stable transformation [158]. EVs derived from glioblastoma cells carrying mutant epidermal growth factor receptor (EGFR) variant III (EGFRvIII) transcripts were found to stimulate tubule formation in ex vivo angiogenesis assays, although the role of EGFRvIII was not explicitly documented in this setting [110]. In another study, transfer of EGFRvIII protein from aggressive to indolent glioma cells triggered elevated production of VEGF in the latter cell population, with a potential to impact their angiogenic phenotype [109]. Furthermore, cancer cell-derived EVs containing oncogenic EGFR were able to transfer this receptor to primary endothelial cells in vitro and in vivo, resulting in the onset of autocrine VEGF production and phosphorylation of VEGFR2 [118].

A more recent analysis of the vascular activity of oncogenic EVs led to the description of vasectasia, a non-angiogenic form of abnormal vascular growth in cancer [60]. In the related study, patient-derived mesenchymal glioma stem cells (GSCs) were found to produce EVs containing oncogenic EGFRvIII, both as oncoprotein and as mutant transcripts. Endothelial cells exposed to these EVs acquired a protracted ectopic expression of the exogenous, phosphorylated and ostensibly bioactive EGFR, along with a dramatic increase in growth and migratory properties in vitro [60]. In mouse orthotopic xenograft models, the content of EGFRvIII in mesenchymal GSCs and their EVs triggered a unique vascular growth pattern involving a circumferential vascular enlargement of intra-tumoural blood vessels, instead of angiogenic capillaries. These large vessels were resistant to treatment with VEGF pathway inhibitors, but were responsive to the EGFR blocking agent Dacomitinib [60]. This EGFRvIII-driven, non-angiogenic endothelial growth response mediated by EVs was referred to as vasectasia, to signify the dilated nature and dysmorphic features of the resulting blood vessels. Single-cell sequencing experiments revealed that vasectasia was associated with an increase in a proliferative subset of endothelial cells at the expense of their angiogenic counterparts, and that phenotypes of tumour-associated endothelial cells were highly abnormal in comparison to the surrounding brain [60]. These observations suggest that the transfer of oncogenic cargo directly to endothelial cells may interfere with their regulatory circuitry and trigger atypical vascular responses resistant to conventional anti-angiogenic agents, a trait consistent with the resistance of high-grade brain tumours to standard antiangiogenic therapy in the clinic [163]. While signalling events participating in vasectasia are being investigated [60], it is conceivable that similar EV-mediated oncogene transfers may occur in relation to other cells across the TME, resulting in more complex vascular and microenvironmental responses.

It is possible that different cancer-causing mutations or oncogenic epigenetic alterations may induce different vascular patterns through activities of oncogenic EVs and/or soluble angiogenic factors. The case in point is the proneural subtype of GSCs. Upon orthotopic implantation of these cells, the emerging brain tumours contain dense angiogenic networks of capillary blood vessels rather than vasectasia [60]. Proneural GSCs are devoid of oncogenic EGFR expression, but may contain other transforming mutations, such as those affecting PDGFRA, CDKN2A, IDH1 and epigenetic modifiers [164,165]. Proneural GSCs produce copious amounts of soluble VEGF, which is not incorporated into EVs [60]. Instead, these EVs exhibit unique protein profiles, and these change dramatically upon serum-induced differentiation affecting the epigenome [155]. Indeed, oncogenic changes in the epigenome represent a poorly understood aspect of cancer-driven vascular remodelling and EV-mediated communication. Of interest, a recent study examined the vascular consequences of two different epigenome-altering oncohistone mutations H3K27M and H3G34R in a pediatric high-grade glioma model. These mutations occurring against the background of other oncogenic alterations triggered the growth of brain tumours with dramatically different vascular responses including either angiogenesis-like, or dilated (vasectasia-like) vascular patterns, respectively. These tumours also emerged in different locations in the brain [166]. Whether EV-related mechanisms were involved in this case remains unknown, but is of great interest.

## 10. Particulate Mediators of Tumour–Vascular Communication in Cancer

Direct transfer of oncogenes represents but one mechanism through which particulate secretome can impact vascular functions in cancer. It is noteworthy that even cancer cells that are uniformly positive for oncogenic proteins incorporate them into only a fraction of their derived EVs [149], while the role of other cancer-derived EV subpopulations remains poorly studied. Moreover, cancer-derived EVs may be enriched with canonical mediators of vascular regulation including angiogenesis, as recently reviewed [15,167,168,169] (Table 1).

**Table 1:** Examples of extracellular vesicle (EV)-associated mediators involved in vascular effects impacting cancer dissemination and metastasis.

Categories	EV Molecules	Cellular Origin	Effect on Vasculature	References
**Angiogenesis** (tumour EVs)	VEGF	Human umbilical vein endothelial cells (HUVECs) and human ovarian carcinoma A2780 cells	Promotes pro-angiogenic effect	Taraboletti, G. et al. (2006) [130]
	VEGF	Glioblastoma cells	Promotes brain microvascular endothelial cell proliferation and migration in vitro	Skog, J. et al. (2008) [110]
	EGFRvIII	U373 (human astrocytoma) cells and U373-expressing EGFRvIII	Promotion of elevated VEGF secretion	Al-Nedawi, K. et al. (2008) [109]
	EGFR	Epidermoid carcinoma, lung adenocarcinoma and colorectal adenocarcinoma	Activation of MAPK and AKT signalling in endothelial cells and promotion VEGF autocrine signalling	Al-Nedawi, K. et al. (2009) [118]
	VEGF-A	Glioblastoma stem-like cells	Induction of brain EC permeability and angiogenesis	Treps, L. et al. (2017) [120]
	EPHB2	Head and neck squamous cell carcinoma (OSC19, Detroit 562, SCC61, MOC1 and MOC2)	Induction of tumour angiogenesis by ephrin-B reverse signalling such as STAT3 phosphorylation (but without affecting phosphorylated VEGFR2)	Sato, S. et al. (2019) [170]
	Delta-like 4	HUVECs and U87GM cells	Promotion of tip cell phenotype and endothelial tube formation	Sheldon, H. et al. (2010) [132]
	GGGU motif-containing miRNAs	HEK293T and CAL27 cells; oral squamous cell carcinoma (OSCC) patient tissues	EGFR boosts PCBP2 expression via transcriptional regulation, which then promotes the loading of specific miRNAs into sEVs by binding to the “GGGU” motif, thereby driving tumour angiogenesis	Xia, H. F. et al. (2025) [171]
	miR-210	Breast cancer cells	Promotion of endothelial cell activation and cancer metastasis	Kosaka, N. et al. (2013) [172]
	miR-182-5p	Breast cancer cells (MDA-MB-231)	Promotes the proliferation, migration, and angiogenesis of HUVECs in vitro and in vivo; promotes tumourigenesis and metastasis of breast cancer cells by regulating the CMTM7/EGFR/AKT signalling axis .	Lu, C et al. (2021) [173]
	miR-205	Ovarian cancer cells	Promotes angiogenesis via the PTEN-AKT pathway and induces tumour metastasis	He, L. et al. (2019) [174]
	FLT1	Endothelial and nasopharyngeal carcinoma (NPC) cells	Induces a positive feedback loop between NPC cells and endothelial cells to promote tumour angiogenesis and tumour metastasis via PI3K/AKT pathway	Li, F. et al. (2025) [175]
	TGFb	Head and neck squamous cell carcinoma cells	TGFb-positive cancer EVs reprogram macrophages to proangiogenic phenotype	Ludwig, N. et al. (2022) [176]
	miR-155-5p	Melanoma cells (B16F10)	Melanoma EVs trigger proangiogenic switch in CAFs	Zhou et al. (2018) [177]
**Angiogenesis** (stromal EVs)	VEGF, HGF, ANG-1	Bone marrow-derived fibroblasts from multiple myeloma patients	Fibroblast-derived EVs were enriched in angiogenesis-regulating factors and impacted endothelial cells in uptake-dependent and independent manner	Lamanuzzi et al. (2022) [178]
	miR-21-5p	Macrophages from head and neck squamous cell carcinoma (SCC25)	EVs from tumour-associated macrophages carry miR-21-5p to endothelial cells regulating YAP1/HIF1a pathway	Yan et al. (2024) [179]
	HOXD11	Cancer-associated fibroblasts (CAFs) from ovarian carcinoma	CAF-EV-associated HOXD11 regulated FN1 and angiogenesis in ovarian cancer xenografts	Chen et al. (2025) [180]
**Non-angiogenic vascular growth**	EGFR/EGFRvIII	Glioma stem cells (mesenchymal subtype)	Promotion of vasectasia (a novel neovascularization characterized by increased circumferential blood vessel growth)	Spinelli, C. et al. (2024) [60]
	Cell migration-inducing and hyaluronan-binding protein (CEMIP)	Breast cancer cells and brain metastatic slices	Induces vascular remodelling, inflammation and vascular co-option, thereby promoting brain metastasis	Rodrigues, G. et al. (2019) [181]
**Endothelial barrier modification**	miR-181c	Brain metastatic breast cancer cells and breast cancer patient sera	Promotion of blood–brain barrier destruction by altering tight junction proteins ZO-1, occludin and claudin-5	Tominaga, N. et al. (2015) [35]
	A disintegrin and metalloproteina-se 17 (ADAM17)	Colorectal cancer cells (HCT116) and patient plasma	Disrupts endothelial cell barrier and enhances vascular permeability by influencing vascular endothelial cadherin (VE-cadherin) cell membrane localization, thereby promoting lung and liver metastases	Li, K. et al. (2024) [182]
	Clathrin light chain A (CLTA)	Hepatocellular carcinoma cells and patient sera	Disrupts vascular endothelial barrier integrity by stabilizing basigin (BSG/CD147) to remodel microvascular niche and enhance pulmonary vessel leakage, thereby promoting liver and lung metastases	Xu, Y. et al. (2023) [183]
**Thrombosis/coagulation**	Podoplanin, tissue factor	Glioblastoma cells	Promotion of thrombosis	Tawil, N. et al. (2021) [30]
	Tissue factor	Colorectal cancer cells	Mediator of cancer-associated coagulation, promotion of angiogenesis	Yu, J. L. et al. (2005) [127]
**Others**	Matrix metalloproteinases	HUVECs	Angiocrine reprogramming of glioma stem cells, contributing to tumour aggressiveness and therapy resistance	Adnani, L. et al. (2022) [19]
	Fibronectin	HT1080 fibrosarcoma cell line	Promotion of cell adhesion and directional cell movement	Sung, B. H. et al. (2015) [129]
	NGFR	Melanoma cells and patient tissue samples	Promotion of lymphangiogenesis and lymph node metastasis	García-Silva, S. et al. (2024) [184]

For example, in certain cancer settings, EVs may contain measurable amounts of VEGF, which could be released as bursts near areas of increased TME acidity, thereby creating potentially pro-angiogenic conditions [130]. Moreover, tumour hypoxia may impact the functional cargo of EVs released from cancer cells [185]. Some GSCs were reported to shed EVs containing bioactive VEGF [120], but other reports suggested that GSCs secrete VEGF mainly in a soluble form [60]. EVs from head and neck squamous cell carcinoma (HNSCC) were recently described as carriers of ephrin type B receptor 2 (EPHB2), which upon contact with endothelial cells activated cell-associated ephrin B2-mediated effects, a fascinating case for ‘reverse’ signalling by EPHB2 ligands leading to angiogenesis-like responses [170]. Ephrin B2 signalling is involved in neuronal guidance and juxtacrine arterial differentiation of endothelial cells, as well as embryonal angiogenesis [186]. Similarly, DLL4, another cell-associated ligand interacting with NOTCH receptor, was described as cargo of endothelial and cancer EVs [132]. Such DLL4-loaded EVs were able to induce tip cell phenotypes in endothelial cells and promote endothelial tube formation in vivo [132].

Cancer EVs are also a source of multiple coding and non-coding RNA biotypes [187] that can be transferred to endothelial cells and elicit biological responses [168]. For example, glioma-derived EVs containing multiple species of microRNA were able to stimulate angiogenic responses and changes in endothelial gene expression profiles, which were distinctively different than those triggered by canonical angiogenic factors [188]. In one study, the transfer of EV-associated EGFRvIII mRNA was shown to be sufficient to induce endothelial growth responses [60], while in another study, EGFR expression in cancer cells triggered upregulation of poly(rC)-binding protein 2 (PCBP2), which enhanced loading of pro-angiogenic microRNA into EVs [171]. These and other recently reviewed examples [168,189] highlight the diverse roles of EVs as intercellular carriers of blood vessel regulating RNA. Similarly, EV-mediated transfer of cancer DNA was reported to occur between tumour cells and endothelium [156,190,191] but the contribution of this process to vascular responses in cancer and metastasis is not fully understood. Some of the elements/examples of EVP interactions with the angiogenesis and vascular regulatory networks are depicted in Figure 3.

The dynamic between vascular system and cancer cell populations can also entail more complex EVP-mediated communication networks [192,193]. For example, recruitment of tumour-associated macrophages (TAMs) through the release of TGFb-positive EVs may result in macrophage-dependent induction of angiogenesis [176]. TAMs may also deploy EVs with more direct pro-angiogenic activity, including transfer of miR-21-5p to endothelial cells [179]. Moreover, myeloid cells may also form pro-thrombotic niches where EVs carrying beta-2 integrin engage with a platelet-dependent micro-thrombotic and pro-metastatic mechanism in lungs [32]. There is also an extensive body of published work suggesting multiple EV-dependent mechanisms through which cancer-associated fibroblasts (CAFs) could contribute to tumour angiogenesis and endothelial cell responses [177,178,180,194,195,196], as extensively reviewed elsewhere [192]. Some of these effects are exemplified by references included in Table 1 (e.g., [173,174,175,181,182,183] and others).

The role of EVPs in tumour–vascular interaction also includes angiocrine effects [19,197]. While it has long been recognized that endothelial cells may modify the properties of adjacent cancer cells [18], including by enhancing their stemness [21], a recent study suggested that these influences may also reprogram the subtype of cancer stem cells [19]. In this setting, endothelial cell-derived EVs were shown to outcompete autocrine EVs released by proneural glioma stem cells resulting in disruption in their neurosphere-forming ability coupled with down-regulation of the NOTCH pathway and expression of mesenchymal-like, pro-invasive phenotypes [19]. Whether endothelial EVs have a role in other cancer contexts, locally or systemically, including during metastasis, is presently unclear, but is of considerable interest.

## 11. Extracellular Vesicles as Mediators of Pre-Metastatic Niche Formation

Among the most intriguing aspects of EVP involvement in the development of metastatic disease is their impact on the vasculature at regional lymph nodes and at distant organ sites, including sites of impending metastatic colonization [5,143,198]. These sites are often described as pre-metastatic niches (PMNs) and involve activation of resident cells [74,88,199,200,201], recruitment of myeloid cells [87,88], alteration to the endothelial lining [35], and/or focal deposition of microthrombi [32,89], all of which increase the probability of subsequent cancer cell homing and formation of secondary tumour deposits. These effects could either increase the overall metastatic dissemination rate [88] or result in pro-metastatic conditioning of specific organ sites (e.g., lung, liver or brain) by EVs with organ-specific homing patterns, usually originating from cancer cells exhibiting the corresponding metastatic tropism [74].

Cancer cells with increased metastatic potential may possess an intrinsic ability to colonize distant organ sites [75,202], even without prior systemic conditioning by EVPs, or other factors. This is evident from experiments involving direct intravascular delivery of cancer cells in the absence of a primary tumour or any other source of tumour EVs [202]. However, the state of systemic homeostasis [5], host genetic factors [203], tissue injury (including iatrogenic) [204,205] and other systemic effects undoubtedly impact the scale of metastatic dissemination. While these factors tend to be considered as mostly pro-metastatic, it is important to keep in mind that EVP-mediated systemic effects may also have anti-metastatic consequences. For example, cancer EVP-induced liver steatosis correlates with a low rate of liver metastasis [9]. Similarly, stimulation of innate immunity in the liver by EV-associated chromatin [6,206] acts as an anti-metastatic mechanism [6]. Moreover, in one study, EVs released by non-metastatic cancer cells exerted a systemic anti-metastatic effect via modulation of myeloid cells [12].

Among the multiple cell types involved in pro-metastatic systemic effects of cancer EVPs [5,207], the vascular compartment plays a particularly central role. Indeed, endothelial and myeloid cells are the principal receptacles for circulating EVs [208], and may serve as reservoirs or targets of their cargo [156,209]. Along these lines, circulating cancer EVPs have been implicated in triggering damage to endothelial lining coupled with increased vascular permeability in organs prone for metastasis [88]. Several mechanisms may play a role in such responses including the delivery of microRNA-181 [35] and other processes [210,211]. Whether EVPs known to be involved in endothelial damage in cancer-unrelated vascular conditions [212,213] may also play a similar role when superimposed with cancer still remains to be investigated. Similarly, dramatic effects on immune cell exclusion from the TME following genetic perturbations within elements of the EV biogenesis machinery, such as Rab27a/b proteins, are of great biological and therapeutic interest [214]. EVs are also potential triggers of microthrombosis and such changes have been detected in the lungs of cancer-bearing mice where they were implicated in increased formation of lung metastases [32,89].

EV-associated miR-210 was reported to contribute to increased efficiency of lung metastasis through stimulation of angiogenic activity of endothelial cells [172]. In another study, EVs released from the primary tumour were found to condition regional lymph nodes for metastasis [28]. Subsequent analyses of this response revealed that nerve growth factor receptor (NGFR/p75NTR) was released as cargo of EVs from metastatic melanoma cells and triggered lymphangiogenic responses, contributing to lymph node metastasis. This receptor transfer process resulted in activation of several signalling pathways, leading to the expression of adhesion molecules (ICAM-1) by lymphatic blood vessels [184]. It is possible that EVs may contribute to the release of endothelial cells from quiescence, which in turn may facilitate the metastatic growth of already disseminated dormant cancer cells [79].

Conversely, cancer-related EVs may also curtail vascular responses, angiogenesis and metastatic progression. The case in point is the aforementioned recent study where EVs released systemically from non-metastatic cancer cells were found to reprogram patrolling monocytes, leading to upregulation of pigment epithelium-derived factor (PEDF), which is a potent inhibitor of angiogenesis. Whether EVs are also involved in previously reported cases of metastasis inhibition by the primary tumour mass (‘concomitant immunity’) [86] is presently unclear, but the systemic release of angiogenic inhibitors has been implicated in some of these studies [215]. It is tempting to speculate that some of these inhibitors could be associated with circulating EVs [211].

## 12. Therapeutic and Diagnostic Implications of EVP-Dependent Vascular Checkpoints in Metastatic Cancer—A Perspective

Recent efforts to understand the regulation of the vascular system by the particulate secretome in metastatic cancer unveiled several mechanisms that could be exploited for therapeutic purposes. EVP biogenesis and cargo loading are complex processes with multiple targetable regulatory nodes, such as the formation of EVP precursors in the endosome or plasma membrane, packaging effector molecules into EVPs, release of EVPs from cells, their uptake by target cells and the resulting responses [5,95,159]. Moreover, different populations of EVPs may compete for target cells [19], thereby creating a possibility of designing ‘decoy’ EVs to interfere with the processes in question. EV pathways are also amenable to exploitation in the quest for EV therapeutics or drug carriers with unique biological and pharmacological properties, as extensively reviewed elsewhere [141,216]. Naturally, these efforts could be targeted at various aspects of the metastatic process including the vascular checkpoints. Similarly, the content of circulating EVs is potentially informative about the ongoing vascular responses to cancer and could be adapted as a component of liquid biopsy diagnostics, i.e., to anticipate the formation of premetastatic niche, thrombosis or other events [5,199,217].

While these are promising and hopeful prospects, the current research landscape also reveals the emerging challenges. For example, still very little is known about the premetastatic niche biology in specific human cancers, or about EVP-mediated vascular responses in pediatric solid tumours, which are also relatively understudied in the context of EVP therapeutics and EVP-based liquid biopsy [218]. In a more general sense, the analysis of the impact that EVPs may have on the vasculature is still (for understandable reasons) often driven by experimental model systems rather than characteristics of specific human diseases, their subtypes, dynamics and molecular complexity, as well as their biological and cellular heterogeneity, all increasingly apparent from single-cell sequencing studies [219].

Correspondingly, while the heterogeneity of EVPs is widely acknowledged [220], its origin, extent, nature, and diagnostic value are not fully incorporated into therapeutic and diagnostic paradigms in cancer, including the impact on the vasculature and metastasis [102]. In this regard, EVP landscapes are as complex as (or perhaps more so than) the landscapes of the underlying cancer and stromal cells and so are their effects on their cellular targets. This is relevant as EV subsets may compete or cooperate with one another and interact with other cell–cell communication pathways (e.g., growth factors or junctions). Indeed, EVs released from endothelial cells compete with autocrine cancer EVs in regulating the behaviour of glioma stem cells [19], and this is likely one of many similar (competitive or cooperative) scenarios involving particulate mediators.

The effects of oncogenic EVs in molding the vascular architecture in tumours is not isolated from that of soluble angiogenic factors and inhibitors [60]. These responses may vary in different tissues and cancer contexts, as do their associated endothelial cells and their surrounding microenvironments [66]. It is possible that while in certain settings, the effects of EVPs on the vasculature and metastasis may be critical, in other settings, they may be negligible, and this is a very important distinction that still needs to be defined and properly contextualized. Therefore, much work lies ahead, and the EVP paradigm will undoubtedly inspire future studies on tumour–vascular interactions in metastasis.
